# Occult hepatitis B in patients with cancer during immunotherapy with or without chemotherapy: A real-life retrospective single-center cohort study

**DOI:** 10.3389/fonc.2023.1044098

**Published:** 2023-01-24

**Authors:** Angioletta Lasagna, Giuseppe Albi, Renato Maserati, Andrea Zuccarini, Mattia Quaccini, Fausto Baldanti, Paolo Sacchi, Raffaele Bruno, Paolo Pedrazzoli

**Affiliations:** ^1^ Medical Oncology Unit, Fondazione Istituto di Ricerca e Cura a Carattere Scientifico (IRCCS) Policlinico San Matteo, Pavia, Italy; ^2^ Division of Infectious Diseases I, Fondazione Istituto di Ricerca e Cura a Carattere Scientifico (IRCCS) Policlinico San Matteo, Pavia, Italy; ^3^ Microbiology and Virology Department, Fondazione Istituto di Ricerca e Cura a Carattere Scientifico (IRCCS) Policlinico San Matteo, Pavia, Italy; ^4^ Department of Clinical Surgical Diagnostic and Pediatric Sciences, University of Pavia, Pavia, Italy; ^5^ Department of Internal Medicine and Medical Therapy, University of Pavia, Pavia, Italy

**Keywords:** occult hepatitis B, cancer, immunotherapy, reactivation, screening, real-life study

## Abstract

**Introduction:**

Few data about the safety of immune checkpoint inhibitors (ICIs) in the patients with solid tumor with Occult Hepatitis B Virus (OBI) are available. According to the Taormina Workshop on Occult HBV Infection Faculty Members we defined as potential-OBI (pOBI) the HBV DNA negativity with anti-hepatitis B core antibody (anti-HBc) positivity (pOBI seropositive), and the patients with HBsAg–negative and anti-HBc–negative and Hepatitis B surface antibody (anti-HBs)–negative are defined pOBI seronegative. The aim of this study is to investigate the prevalence of OBI in patients with solid tumors undergoing ICIs with or without chemotherapy and the incidence of reactivation (HBVr).

**Methods:**

We retrospectively enrolled all HBsAg negative subjects who had received ICIs for at least three months. HBsAg and HBV DNA levels were repeated every 3 months until the end of the study and/or in case of ALT alterations. A univariate analysis was conducted in order to study for each variable available its ability to distinguish a potential OBI seropositive patient from a seronegative one.

**Results:**

150 patients in our Oncology Unit were eligible. One hundred and seventeen patients (78%) received ICI as monotherapy, whereas 33 patients (22%) were treated with chemo-immunotherapy. The mainly used drugs for the ICI monotherapy were Pembrolizumab (47%), Nivolumab (33%) and Atezolizumab (11%). The prevalence of pOBI seropositive patients was 25.3%. We did not observe alterations of liver biochemistry nor HBVr.

**Discussion:**

This study highlights that about a quarter of our population had a potential occult hepatitis B. Immunotherapy might be considered as low risk of reactivation, regardless of the potential presence of episomal covalently closed circular DNA (cccDNA) in the liver, but the correct management still represents a challenge for oncologists and hepatologists.

## Introduction

The currently available therapies for Hepatitis B Virus (HBV) infection are not able to eradicate it and HBV remains a major health problem worldwide (1). According to the WHO estimates, approximately 250 million subjects live with a chronic HBV infection (2). HBV is a prototypical member of the Hepadnaviridae family and after the viral entry in the hepatocytes, the relaxed circular DNA (RC-DNA) is released in the nucleoplasm and converted into an episomal covalently closed circular DNA molecule (cccDNA) (3). cccDNA is fully replication-competent and plays the role of a template for viral transcripts (4). Occult Hepatitis B Infection (OBI) is defined by the presence of cccDNA in hepatocytes and/or HBV DNA in peripheral blood of subjects who are hepatitis B surface antigen (HBsAg) negative (5). A systematic review estimated a prevalence of OBI in Western countries ranging from 19% to 51% (6). The extreme variability in the prevalence of OBI may depend on diagnostic challenges: the gold standard is the detection of HBV DNA in the liver by a biopsy, but it is an invasive procedure without available standardized assays for HBV DNA detection in the liver. Among individuals with OBI, the prevalence of detectable HBV DNA in serum/plasma, used as surrogate, varies depending on the population studied, the sensitivity of the assay used, and whether blood samples at one or more time-points are tested. Many studies have found that HBV DNA is only intermittently detected in serum/plasma, and when detectable, the concentration is low, usually less than 200 IU/ml (about 1,000 copies/ml). The occult HBV infection should be detected by nucleic acid tests (NAT) in blood samples or liver tissue, but in the clinical practice, according to the Taormina Workshop on Occult HBV Infection Faculty Members (5), it is usually defined as potential-OBI (pOBI) the HBV DNA negativity with anti-hepatitis B core antibody (anti-HBc) positivity (pOBI seropositive), and the patients with HBsAg–negative and anti-HBc–negative and Hepatitis B surface antibody (anti-HBs)–negative are defined pOBI seronegative. The clinical significance of HBV infection includes the risk of reactivation in immunocompromised hosts with fulminant hepatitis. There is a high and well-known risk of HBV reactivation (HBVr) in patients with OBI and hematologic malignancies undergoing both treatment with anti-CD20 antibodies and stem-cell transplantation (7, 8), while this risk is more difficult to estimate among patients with solid tumor due to the scarcity of available data, in particular during immune checkpoint inhibitors (ICIs) treatment (9). Based on the Asian-Pacific Association for the study of the liver (APASL) clinical practice guideline on HBVr, there is a High risk of reactivation (> 10%) with ICIs (moderate to high risk) if patients have HBsAg (+), but this risk is defined Uncertain if patients have HbsAg(-)/anti-Hbc (+) (10). So far, the prevalence of OBI and risk of reactivation in this setting remain unclear. According to the ASCO Provisional Clinical Opinion (PCO), patients with OBI undergoing ICIs should be monitored with HBsAg and ALT testing every 3 months (with subsequent HBV DNA testing if a hepatitis flare develops) and the antiviral therapy is limited to seroreversion of HBsAg or if HBV DNA exceeds 1,000 IU/mL (11). However, the optimal follow-up schedule in OBI patients has not been defined yet, nor the need for any prophylactic therapy.

The aim of this study is to investigate the prevalence of OBI in patients with solid tumors undergoing immunotherapy with or without chemotherapy and HBVr during the therapy.

## Materials and methods

### Study design and setting

This was a real-life retrospective single-center cohort study. Between June 2020 and August 2021, patients with solid tumors who had HBsAg negativity and had received immunotherapy with or without chemotherapy for at least three months were retrospectively reviewed. We included all the types of cancer, with the only exception of hepatocellular carcinoma (HCC). All the types of immunotherapy (anti cytotoxic T-lymphocyte antigen-4 (CTLA-4) or anti-Programmed cell death protein 1 (anti-PD-1) or anti-Programmed Cell Death Ligand 1 (anti-PD-L1) or a combination of anti-CTLA-4 and anti-PD1) with or without chemotherapy were accepted. The patients were excluded if they had other positive viral markers including Immunoglobin M (IgM) antibody to hepatitis A virus, antibody to hepatitis C virus (HCV), Immunoglobin G (IgG) antibody to hepatitis D virus, IgM antibody to hepatitis E virus, or antibody to human immunodeficiency virus, or with other cause of liver disease with known etiology (autoimmune liver disorder and storage liver disease), under prophylaxis for HBV and vaccinated for hepatitis B.

We ruled out the HBV-HCV co-infection because it might be a confounding factor. It is well that one virus may be typically dominant over the other. In the case of a HCV dominant coinfection, HCV is able to suppress the HBV replication and a proportion of cases of HCV dominant may have occult HBV infection. Moreover, the HBV-HCV co-infection may decreased HBV DNA levels compared to the monoinfection (12). Therefore, we decided to exclude all the other types of hepatitis.

According to the Taormina Workshop on Occult HBV Infection Faculty Members (5), we defined pOBI as HBV DNA negativity with anti-HBc positivity (pOBI seropositive). The patients with HBsAg–negative and anti-HBc–negative and anti-Hbs–negative were defined “seronegative”.

According to the American Association for the Study of Liver Diseases (AASLD) 2018 hepatitis B guidance we defined HBVr: HBsAg seroreversion and/or an increase of serum HBV DNA by at least 1 log above the lower limit of detection of the assay in a patient who had previously undetectable HBsAg and HBV DNA in serum, and a more than 1 log increase in serum HBV DNA in people who had detectable HBV DNA at baseline (13).

The clinical outcomes analyzed in this study were the evaluation of the prevalence of OBI in cancer patients on immunotherapy at inclusion in the study and the evaluation of the incidence of HBVr during the therapy throughout the duration of the study.

### Data collection

Tests for serum HBsAg, HBsAb, HBcAb, hepatitis B e antigen (HBeAg), hepatitis B e antibody (HBeAb) were performed at baseline such as HBV DNA testing. HIV and HCV screening were also done at baseline. pOBI repeated liver function tests every cycle of immunotherapy.

HBsAg and HBV DNA level were repeated every 3 months until the end of the study and/or in case of alanine aminotransferase (ALT) alterations, as recommended by the Associazione Italiana Per lo Studio del Fegato (AISF) (14).

Serum HBV DNA was assessed using quantitative real-time PCR using Abbott m2000rt platform (Abbott, Germany) following the manufacturer’s instructions. The lowest limit of HBV viral load of the PCR was 15 IU/ml.

Hepatitis flare was defined as serum ALT level >3 × upper limit of normal (ULN) or an ALT increase >100 U/L, and severe hepatitis was defined as an ALT increase >10 × ULN or Total Bilirubin (TN) >1.5 × ULN.

This study was conducted according to the Strengthening the Reporting of Observational Studies in Epidemiology (STROBE) Statement for reporting observational studies (15) and was approved by the local Ethics Committee (Comitato Etico Area Pavia) and Institutional Review Board. All the subjects signed an informed written consent.

### Statistical analysis

The patients’ characteristics are described as median and interquartile range (Shapiro-Wilks test excluded the normal distribution hypothesis) if quantitative variables. Qualitative variables are described as count and percentages. With 150 subjects we have estimated the prevalence of OBI with a precision (half-width of 95% confidence interval 95%CI) of 7% if prevalence is around 20%.

We have reported the prevalence of potential seropositive OBI patients with binomial exact 95% CI.

A univariate analysis was conducted in order to study for each variable available its ability to distinguish a potential OBI seropositive patient from a seronegative one. We used a non-parametric test (U-Mann-Whitney) for comparing quantitative variable and the Chi-square test with Yates correction for comparing qualitative variable , as the total number of frequencies of the qualitative variables compared is <200.

In addition, for the qualitative variables with 3 or more levels, that show a significant pvalue, we have performed a *post-hoc* test with Bonferroni correction in order to test which groups were different.

All statistical tests were two-sided and a pvalue ≤ 0.05 was considered statistically significant.

We have performed statistical analysis with R statistical environment (R Foundation for Statistical Computing V4.0.5, https://www.r-project.org/.)

## Results

### Characteristics of the study population

Out of 163 patients in our Medical Oncology Unit over the study period, 150 were eligible. Five patients were excluded for HBV vaccination, one patient was under prophylaxis for HBV (lamivudine), four patients were HBsAg positive and 3 were positive for anti-HCV antibody. HBV viral load result was available for all of the study participants. Nobody had detectable HBV DNA viral load. The patients’ characteristics are summarized in **Table 1**. The median age was 67.5 years (IQR: 15.7; range: 14–81). One hundred and five (70%) patients were male. The main tumor types were non-small cell lung carcinoma (NSCLC; n = 96, 64%) and melanoma (n = 31, 21%). One hundred and seventeen patients (78%) received ICI as monotherapy, whereas 33 patients (22%) were treated with chemo-immunotherapy. The mainly used drugs for the ICI monotherapy were Pembrolizumab (47%), Nivolumab (33%) and Atezolizumab (11%). At baseline and during the follow up period, no patients had received steroid therapy.

**Table 1:** Patients’ characteristics.

Sample size = 150	
Age	67.5 (15.75) median
< 50	6 (4%)
≥ 50	144 (96%)
Sex	
Female	45 (30%)
Male	105 (70%)
Tumor type	
Lung	96 (64%)
Melanoma	31 (20.7%)
Kidney	4 (2.7%)
Head & Neck	13 (8.6%)
Other	6 (4%)
Disease stage	
III	19 (12.7%)
IV	131 (87.3%)
Disease line	
Adjuvant Maintenance	14 (9.3%) 8 (5.3%)
1^st^ line	76 (50.7%)
2^nd^ line	52 (34.7%)
Immunotherapy type	
Pembrolizumab	70 (46.7%)
Nivolumab	49 (32.7%)
Avelumab	2 (1.3%)
Cemiplimab	–
Durvalumab	8 (5.3%)
Ipilimumab	4 (2.7%)
Atezolizumab	17 (11.3%)
Association to chemotherapy	
Yes	33 (22%)
No	117 (78%)
Hepatic biopsy	
Yes	1 (0.65%)
No	149 (99.35%)
Abdomen ultrasound	
Yes	3 (2%)
No	147 (98%)
Immunotherapy	
Ongoing	84 (56%)
Stop for exitus	57 (38%)
Stop for progression disease	7 (4.7%)
Stop as scheduled	2 (1.3%)

The virologic HBV status of the 38 pOBI seropositive patients and of the 112 seronegative patients was detailed and outlined by subsets in **Table 2**.

**Table 2:** Seromarker distribution.

Seromarkers	Num patients (%)
**Anti-Hbc+ (total)**	**38 (25.3%)**
Anti-HBc+/anti-HBs+/anti-HBe+	10 (6.6%)
Anti-HBc+/anti-HBs+/anti-HBe−	19 (12.6%)
Anti-HBc+/anti-HBs−/anti-HBe+	3 (2%)
Anti-HBc+/anti-HBs−/anti-HBe−	6 (4%)
**Anti-Hbc− (total)**	**112 (74.7%)**
Anti-HBc−/anti-HBs+/anti-HBe+	0
Anti-HBc−/anti-HBs+/anti-HBe−	0
Anti-HBc−/anti-HBs−/anti-HBe−	112 (74.7%)
Total	150

anti-HBs, anti-hepatitis B surface antibodies; Anti-HBc, anti-hepatitis B core antibodies; anti-HBe, anti-hepatitis B envelope antibodies.

### Trend of liver biochemistry

During the follow up, we did not observe alterations of liver biochemistry. Only two patients showed a slightly increase of ALT value respect to the baseline (one subject pOBI seropositive and one seronegative) during the first three months.

Only 98 patients (65.3%) were still undergoing therapy with ICIs at a 6-month follow-up: nobody developed hepatic flare in the further follow-up period. At the end of follow up period (+ 12 months), only 90 patients (60%) were still undergoing therapy with ICIs with no HBVr and/or hepatic flare.

### pOBI seropositive and seronegative comparison

The univariate analysis did not demonstrate significant difference between the two groups according to the following variables: sex, age (discretized in 50 and >50), tumor type, disease stage. Differently, we observed a significant difference between the two groups when considering the median age, the association to chemotherapy, the immunotherapy type and the disease line, as shown in **Table 3**.

**Table 3:** Univariate analysis results.

	pOBI seropositive 38 (25.3%)	Seronegative patients 112 (74.7%)	P value
**Sex**			0.44
Male	29 (76.3%)	76 (67.9%)	
Female	9 (23.4%)	36 (32.1%)	
**Age**			1
≤ 50	1 (2.6%)	5 (4.5%)	**0.008**
> 50	37 (97.4%)	107 (95.5%)	
Median age	71 (9.75)	66.5 (15)	
**Tumor type**			0.078
Lung	25 (65.8%)	71 (62.4%)	
Melanoma	5 (13.2%)	26 (23.2%)	
Kidney	−	4 (3.6%)	
Head & Neck	4 (10.5%)	9 (8%)	
Other	4 (10.5%)	2 (1.8%)	
**Disease stage**			0.196
III	2 (5.3%)	17 (15.2%)	
IV	36 (94.7%)	95 (84.8%)	
**Association to chemotherapy**			**0.002**
No	1 (2.6%)	32 (28.6%)	
Yes	37 (97.4%)	80 (71.4%)	
**Immunotherapy type**			0.032*
Pembrolizumab	10 (26.3%)	60 (53.6%)	
Nivolumab	20 (52.7%)	29 (25.9%)	
Avelumab	1 (2.6%)	1 (0.9%)	
Cemiplimab	−	−	
Durvalumab	1 (2.6%)	7 (6.2%)	
Ipilimumab	1 (2.6%)	3 (2.7%)	
Atezolizumab	5 (13.2%)	12 (10.7%)	
**Disease line**			0.002**
Adjuvant	2 (5.3%)	12 (10.7%)	
Maintenance	1 (2.6%)	7 (6.2%)	
1^st^ line	12 (31.6%)	64 (57.2%)	
2^nd^ line	23 (60.5%)	29 (25.9%)	
**Immunotherapy**			0.711
Ongoing	21 (55.3%)	63 (56.2%)	
Stop for exitus	16 (42.1%)	41 (36.6%)	
Stop for progression disease	1 (2.6%)	6 (5.4%)	
Stop as scheduled	–	2 (1.8%)	

(*) The only statistically significant pairwise comparison was between Pembrolizumab and Nivolumab with P value = 0.021.

(**) The only statistically significant pairwise comparison was between 1^st^ line and 2^nd^ line with P value = 0.003.

P values are reported in bold if significant.

In particular, the pOBI seropositive group was characterized by a higher percentage of patients treated with Nivolumab and a lower percentage of patients treated with Pembrolizumab (p=0.021), and by a higher percentage of patients in a 2nd disease line (p=0.003), compared to the seronegative patients.

### Incidence of reactivation

During the study period, we did not observe any HBVr. Only one pOBI seropositive patient showed an increase of ALT (> 3 x ULN) and a transient detectability of HBV-DNA. His baseline virologic HBV status was anti-Hbc+, anti-Hbs+ and anti-Hbe−.

The abdominal ultrasound was unremarkable. The liver histology showed only a portal inflammation, without histologic evidence of HBVr or of a Drug Induced Liver Injury (DILI).

After four cycles of Pembrolizumab, one patient (Anti-Hbc positive, anti-Hbs negative and anti-Hbe negative) presented with >10 nonbloody bowel movements a day and the colon biopsies demonstrated a microscopic colitis (lymphocytic subtype). He started a corticosteroid treatment with high-dose prednisone (1 mg/kg) without antiviral prophylaxis and we did not observed HBVr.

## Discussion

Immunotherapy has become a cornerstone in the treatment of patients with cancer. However, clinical trials have systematically excluded the patients with viral infections, such as HBV, limiting our knowledge about the safety of ICIs among this special population.

Zhang and colleagues published the first systematic analysis of the incidence of HBVr in a cohort of 114 cancer patients undergoing anti-PD-1/PD-L1 therapy with HBsAg-positivity (16). The study demonstrated that six patients (5.3%) developed HBVr and only one of them had received entecavir as antiviral prophylaxis. The authors recommend the initiation of a prophylactic antiviral treatment for those who are seropositive for HBsAg (16).

To our knowledge, few studies have investigated the risk of reactivation specifically in cancer patients with OBI during the immunotherapy (17, 18).

In particular Yoo and colleagues (17) have evaluated that the incidence rates of HBVr of HBsAg-positive patients and HBsAg-negative patients were 1.0% (5/511) and 0.0% (0/2954), respectively.

Oncologists should be aware of the possible severe clinical consequences of reactivation of OBI, such as acute/fulminant hepatitis and/or liver failure, but it is still difficult to realize the impact of OBI. The first challenge is to determine the real prevalence of OBI. As previously reported, the diagnostic gold standard is the detection of HBV DNA by a liver biopsy, but this procedure is not commonly used; thus, the detection of HBV DNA and of anti-HBc in the blood is used as surrogate (5). OBI is usually divided in two sub-categories: seropositive OBI: anti-HBc positive and/or hepatitis B surface antibody (anti-HBs) positive; seronegative OBI, negative for all HBV markers (5). According to the intermittent detection of HBV DNA, and in the absence of a liver biopsy, we decided to follow up all our patients HBsAg negative over time.

In our cohort of patients, the prevalence of pOBI seropositive patients, carrying HBcAb, was 25.3% (95% CI: 0.186–0.331) while the prevalence of the seronegative patients, not carrying HBcAb, was 74.7% (95% CI: 0.669–0.814). The two groups of patients did not differ significantly for the analyzed variables. Importantly, we have not demonstrated any HBVr.

These observations, if confirmed by larger prospective studies, might better define the category of ICIs as low risk of reactivation, regardless of the potential presence of cccDNA.

Burns and colleagues retrospectively reviewed the pharmacovigilance US Food and Drug Administration (FDA) Adverse Events Reporting System (FAERS) database for the development of HBVr reported with ICIs (19). They have reported no HBVr with Ipilimumab and Avelumab, 10 cases (0.43%) with Pembrolizumab, 7 (0.30%) with Nivolumab, 4 (0.17%) with Atezolizumab, and 1 (0.04%) with Durvalumab. This association was significant only for Pembrolizumab (ROR: 2.32, 95% CI: 1.11–4.28, P=0.013), but not for other ICIs.

The few data of HBVr during ICIs might depend on the postulated therapeutic relationship between PD-1/PD-L1 blockade and the management of chronic viral infections (20). Mouse models with chronic HBV and inhibition of the PD-1/PD-L1 axis have suggested reduction in chronic viral persistence thanks to an accelerated activation of intrahepatic T lymphocytes, *in vivo* viral clearance (21). Moreover, in a phase Ib study, Nivolumab was administered to virally suppressed HBeAg-negative patients causing a significant reduction in quantitative HBsAg levels from baseline, suggesting its possible therapeutic role (22).

These observations should not diminish the clinician’s attention about this issue. The risk of HBVr during ICIs can be triggered if patients receive high-dose steroids for immune-related adverse events (IRAEs), because dexamethasone directly suppresses the T cell function, as described by He and colleagues (23).

Another issue to be considered is that the tissue damage of hepatocytes, enhanced by an accelerated immune response by ICIs, might lead to the release of viral particles whose control may be impaired either for lymphopenia or for an inadequate cytotoxic response (19). On the other side, ICIs might cause an exaggerated host inflammatory response on the host immunity, such as immune reconstitution inflammatory syndrome (IRIS) observed in human immunodeficiency virus (HIV) infected patients, and so they may cause the reactivation of a wide range of infections, including HBV (24).

The strength of our data consists in the evaluation of both pOBI seropositive and seronegative patients. This means that ICIs seem not to affect HBVr both in subjects carrying cccDNA and in subjects not carrying cccDNA. Our paper has several limitations. To begin with, the small sample size of patients evaluated and, secondly, its retrospective nature which leads to selection bias. We evaluated the quality of the paper by using the Study Quality Assessment Tools for Observational Cohort and Cross-Sectional Studies developed by the National Heart, Lung, and Blood Institute (NHLBI) (25). We found that the potential confounding factor are related to the small sample size and by the sequential enrolment of the patients whereas the other items are sufficiently satisfied. Finally, a one-year follow-up may not be sufficient, as reactivations have been reported even months after discontinuation of cancer treatment as described in the case of a patient with chronic lymphocytic leukemia (CLL) who developed hepatic failure 30 months post completion of fludarabine, cyclophosphamide and rituximab (FCR) (26). The detection of HBV DNA can indeed be intermittent and following patients at 3 months interval, as suggested by ASCO PCO (11) might not have captured this value.

Another limitation of the study is the HBV DNA LoD of 15IU/ml when approximately 50% of OBI carry less than 20IU/ml viral load. The absence of any liver biopsy and therefore inability of cccDNA detection is another major weakness of the study, but in patients with cancer, the liver biopsy is not common clinical practice.

Anyway, this is a retrospective study based on the real-life follow up available data and the use of molecular ultrasensitive test was not included in the routinary work-up.

## Conclusions

This is the first study to assess the prevalence of OBI in cancer patients receiving immunotherapy without antiviral prophylaxis and the incidence of HBVr. This study highlights that the prevalence of pOBI seropositive is not irrelevant (about a quarter of our population had a potential occult hepatitis) and so the correct management represents a challenge for oncologists. The current world recommendations indicate that the close monitoring of HBV DNA is required in the absence of antiviral prophylaxis. ICIs may modulate HBV transcriptional activity, so, in order to design individual strategies to prevent HBV-related hepatitis during immunotherapy, further large-scale studies are needed to better identify risk factors and define the optimal follow up of HBV monitoring in this population.

## Data availability statement

The raw data supporting the conclusions of this article will be made available by the authors, without undue reservation.

## Ethics statement

The studies involving human participants were reviewed and approved by Ethics Committee (Comitato Etico Area Pavia) and Institutional Review Board. The patients/participants provided their written informed consent to participate in this study.

## Author contributions

All authors listed have made a substantial, direct, and intellectual contribution to the work and approved it for publication.
